# Discovery and characterization of medaka miRNA genes by next generation sequencing platform

**DOI:** 10.1186/1471-2164-11-S4-S8

**Published:** 2010-12-02

**Authors:** Sung-Chou Li, Wen-Ching Chan, Meng-Ru Ho, Kuo-Wang Tsai, Ling-Yueh Hu, Chun-Hung Lai, Chun-Nan Hsu, Pung-Pung Hwang, Wen-chang Lin

**Affiliations:** 1 Institute of Biomedical Informatics, National Yang-Ming University, Taipei, Taiwan; 2 Bioinformatics Program, Taiwan International Graduate Program, Academia Sinica, Taipei, Taiwan; 3 Institute of Biomedical Sciences, Academia Sinica, Taipei, Taiwan; 4 Institute of Information Sciences, Academia Sinica, Taipei, Taiwan; 5 Information Sciences Institute, University of Southern California, Marina del Rey, CA 90292, USA; 6 Institute of Cellular and Organismic Biology, Academia Sinica, Taiwan

## Abstract

**Background:**

MicroRNAs (miRNAs) are endogenous non-protein-coding RNA genes which exist in a wide variety of organisms, including animals, plants, virus and even unicellular organisms. Medaka (*Oryzias latipes*) is a useful model organism among vertebrate animals. However, no medaka miRNAs have been investigated systematically. It is beneficial to conduct a genome-wide miRNA discovery study using the next generation sequencing (NGS) technology, which has emerged as a powerful sequencing tool for high-throughput analysis.

**Results:**

In this study, we adopted ABI SOLiD platform to generate small RNA sequence reads from medaka tissues, followed by mapping these sequence reads back to medaka genome. The mapped genomic loci were considered as candidate miRNAs and further processed by a support vector machine (SVM) classifier. As result, we identified 599 novel medaka pre-miRNAs, many of which were found to encode more than one isomiRs. Besides, additional minor miRNAs (also called miRNA star) can be also detected with the improvement of sequencing depth. These quantifiable isomiRs and minor miRNAs enable us to further characterize medaka miRNA genes in many aspects. First of all, many medaka candidate pre-miRNAs position close to each other, forming many miRNA clusters, some of which are also conserved across other vertebrate animals. Secondly, during miRNA maturation, there is an arm selection preference of mature miRNAs within precursors. We observed the differences on arm selection preference between our candidate pre-miRNAs and their orthologous ones. We classified these differences into three categories based on the distribution of NGS reads. Finally, we also investigated the relationship between conservation status and expression level of miRNA genes. We concluded that the evolutionally conserved miRNAs were usually the most abundant ones.

**Conclusions:**

Medaka is a widely used model animal and usually involved in many biomedical studies, including the ones on development biology. Identifying and characterizing medaka miRNA genes would benefit the studies using medaka as a model organism.

## Background

MicroRNAs (miRNAs) are endogenous non-protein-coding RNAs with ~22 nucleotides in length. They exerts down-regulation ability, either by translation inhibition or by degradation mRNA, on target genes through complementary binding to their 3'-UTR regions [1]. More and more studies have discovered the critical modulation functions of miRNAs in many physiological activities. miRNA genes were also found to exist in a wide variety of organisms, including animals, plants, virus and even unicellular organisms [1,2], which suggests the evolutionary conservation of miRNA genes and miRNA regulation mechanisms [3]. Therefore, a few reports have investigated cross metazoan or bilaterian conservation of miRNAs and discovered unique miRNA evolution conservatios [4-9] .

Establishing a comprehensive miRNA resource in additional organisms would benefit subsequent researches on subsequent miRNA evolution and function. To do so, a prerequisite identification of miRNA gene in model organisms is essential. However, discovery of miRNAs by traditional experimental approaches, including direct cloning, northern blot assay and stem-loop RT-PCR, is not an easy task due to their relatively small size and distinct tissue expression patterns. A systems biology approach would be preferable for large scale validation.

Recently, next generation sequencing (NGS) technologies, including Roche 454, Illumina GA (Genome Analyzer) and ABI SOLiD platforms, emerged as powerful sequencing platforms for genomic and transcriptomic studies. All NGS platforms have good detection sensitivity by a evaluation study [10]. Therefore, NGS technology has been adopted in transcriptome profiling [11,12,13], SNP identification [14,15], genome sequencing [16,17], biomarker detection [18], and so on. Recently, NGS technology was also applied in miRNA identification and profiling studies. Morin *et al* identified 104 novel human miRNA genes and made a list of miRNAs differentially expressed between two embryo cell libraries [19]. Glazov and colleagues discovered 449 new chicken miRNAs and 39 mirtrons [20]. In addition, Wheeler *et al* not only sequenced miRNAs from metazoan genomes but also interrogated evolution status of discovered miRNA genes [4].

Medaka (*Oryzias latipes*) is a useful model organism among vertebrate animals [21,22]. Although widely used in many researches, little information on medaka gene annotation is available thus far. Up to now, there are only 474 medaka protein-coding reference genes reported in RefSeq release 37. In addition, only small number of fish miRNAs was reported comparing with other animal categories. There are only 360, 131, and 132 miRNA entries in *Danio rerio*, *Fugu rubripes*, and *Tetraodon nigroviridis*, respectively [23]. To date, no medaka miRNAs were discovered and reported even if the miRNA collection in miRBase 14.0 has reach up to 10,883 entries. Since it is a model animal widely used for different research purposes and miRNA genes play critical biological activities, identifying miRNA genes on medaka genome would greatly benefit subsequent studies. In this study, we adopted ABI SOLiD platform for medaka miRNA gene identification. In summary, we identified 599 novel medaka pre-miRNAs, many of which were found to encode more than one isomiRs. Discovery of miRNA genes in medaka genome would enhance further understanding of miRNA evolutions and functions in fish and vertebrates.

## Results

### Initial NGS read analysis

We first extracted RNA from medaka and processed them by the small RNA deep sequencing protocol with ABI SOLiD platform. We classified the collected sequence reads into unique reads and tabulated the copy number of each unique read. Our result showed that the copy number distribution of unique reads diverse dramatically. Owing to the large size of our read collection and for improving analysis confidence, we have selected the unique reads with copy number equal or greater than three for subsequent trimming adaptor and genome mapping steps. As shown in Table 1, 113,650 medaka unique reads (accounting for 3,030,172 mappable reads) can be mapped back to 1,054,853 genome loci in medaka genome. This multiple genome location mapping phenomenon could reflect the incompleteness of genome assembly as well as the possibility of repeat elements existed in medaka genome (see Discussion). These mapped genomic loci and their flanking sequences were individually extracted from genome and considered as candidate mature miRNAs and candidate pre-miRNAs for further processing (see Materials and methods).

**Table 1:** Statistics of mappable sequence reads

# mappable reads	3,030,172
# mappable unique reads	113,650
# mappable genmic loci	1,054,853

### Identified miRNAs by SVM pipeline

Each pair of candidate miRNA and candidate pre-miRNA was subject to calculating their values of classification features and then processed by a SVM pipeline [24]. As a result, 1018 medaka candidate miRNAs were classified as positive hits. Further inspecting their genomic coordinates, we found that these candidate miRNAs were encoded by 599 medaka pre-miRNA. Besides, checking their sequence homology with all known miRNAs in miRBase 14.0, we then classified them into the Medaka homolog (Mh) and the Medaka novel (Mn) sets. Mh group denotes miRNAs with sequence homologous to known miRNAs with at most two nucleotide variations. Mn group denotes miRNAs without sequence homologous to known miRNAs. The statistics of candidate miRNAs were listed in Table 2. We identified 254 homologous and 345 novel pre-miRNAs, encoding 593 and 425 mature miRNAs, respectively. Besides, the numbers of reads from these candidate miRNAs show that miRNA reads account for 28.4% ((469,722+389,984)/3,030,172) of all mappable reads.

**Table 2:** Statistics of candidates in different sets

Set	Mh	Mn
# pre-miRNA	254	345
# isomiRNA	593	425
# reads	469,722	389,984

**Figure 1:**
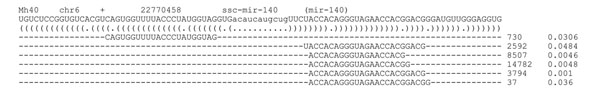
Presentation of candidate information. Each isomiR was generated from independent sequence read and aligned as positive. Copy number denotes the abundance of each isomiR from initial read collection and represents the expression level of each isomiR. p-value denotes the probability at which one candidate was classified as a positive hit by mistake based on the SVM classification model. The term in parenthesis denotes the family to which the orthologous known pre-miRNA belongs.

Many studies demonstrated that one pre-miRNA may encode many mature miRNAs, called isomiRs. The high-throughput advantage of NGS technology enables researchers to perform in-depth analysis on isomiRs. These isomiRs show few sequence variations from the reference miRNA sequences registered in miRBase [4,19,20,25]. In this study, such phenomenon was also observed and one isomiR example was demonstrated in Figure 1. As shown in Figure 1, Mh40 is the 40th candidate pre-miRNAs in Mh set. It is homologous to known ssc-mir-140 so that the orthologous pre-miRNA of Mh40 is ssc-mir-140. Mh40 encoded six isomiRs, one at the 5' arm and five of them at the 3' arm. These six isomiRs came from six independent unique reads, and each of which was independently discovered by the SVM pipeline. Therefore, each of them is tagged with two values, copy number and p-value. Copy number denotes the abundance of each isomiR from initial unique read. And, p-value denotes the probability at which one candidate was classified as a positive hit by mistake based on the SVM classification model. Originally, we used p-value of 0.05 as default threshold of a positive hit. Here, the p-values of all candidate mature miRNAs range from 0.0484 to 0.0003.

Comparing all six isomiRs in Mh40, most variances occur at the 3' end. Although other types of RNA editing were mentioned, e.g. A to G transition catalyzed by adenosine deaminase or C to U transition catalyzed by cytidine deaminase, we believe that they are not significantly prevalent comparing the sequencing errors generated from technology problem [4,19]. Therefore, in this study, we did not consider these RNA editing modifications. We have employed a perfect sequence match mapping criterion and reads with sequence variations were discarded. Besides, 29.2% (175/599) of candidate pre-miRNAs encode isomiRs. Information of all candidates of Mh and Mn sets can be accessed in Additional files 1 and 2.

### miRNA cluster in medaka genome

It is known that many miRNAs are located close to each other and could form a gene cluster [26,27]. miRNA genes in the same cluster might be transcribed from a polycistronic transcript if they were located in a close distance. Based on miRBase’s definition of miRNA cluster (10,000 bp range), we discovered a total 63 miRNA clusters from all candidate pre-miRNAs in medaka genome. The clusters and the candidate pre-miRNAs within them are listed in Additional file 3. As shown in Additional file 3, most clusters have two (41 clusters) or three (11 clusters) pre-miRNAs. These cluster-based pre-miRNAs account for 32.4% (194/599) of all medaka pre-miRNAs. Among all clusters, some clusters have only homologous pre-miRNAs such as cluster 37; some clusters have only novel pre-miRNAs such as cluster 17; and, some clusters have both homologous and novel pre-miRNAs such as cluster 51. In cluster 51, there are two homologous pre-miRNAs at the two ends and five novel pre-miRNAs located within the boundaries of two homologous pre-miRNAs. It is likely that the clustered candidate pre-miRNAs are under the same transcription regulation unit and are more probable to be authentic miRNAs. Therefore, they deserve more attention.

There are six candidate pre-miRNAs, Mh179, Mh180, Mh181, Mh182, Mh183, Mh184, located in cluster 37 at the minus strand of chromosome 21. Their orthologous pre-miRNAs are eca-mir-92a, eca-mir-19b, eca-mir-20a, eca-mir-19a, eca-mir-18a and eca-mir-17, respectively (see Additional file 3). Querying miRBase, we found that these six horse pre-miRNAs also formed a cluster in a reverse order at the plus strand of chromosome 17. Besides, this miRNA cluster is commonly shared by vertebrate animals. This observation indicates these clustered miRNAs could contribute as a conserved transcript unit and evolve together with each other.

### Arm selection preference of mature miRNAs within precursor

During miRNA maturation, there is an arm selection preference of mature miRNAs within precursors. Therefore, most mature miRNAs were generated from either 5’ or 3’ arm of pre-miRNA hairpins. But, it is also observed that some pre-miRNAs could encode mature miRNAs at both arms. By miRBase definition, miRNAs from both arms can be of equal abundance, named with -5p or -3p suffix, or of unequal abundance, named with asterisk(*) suffix for the minor one[3]. In this study, a large number of NGS reads allow us to investigate comprehensively on the arm selection preference of mature miRNA. Based on location preference of mature miRNAs generation, we classified our candidate pre-miRNAs into five categories, including 5P only, 3P only, 5P dominant, 3P dominant, and equal abundance. According to Additional file 1, the five categories account for 43.3%, 41.4%, 6.7%, 5.2% and 3.4%, respectively. Similar distribution was also observed in known Zebrafish miRNAs (data not shown). This distribution pattern shows that most pre-miRNAs encode mature miRNAs mainly at only one side of precursor hairpins.

In a previous report, Wheeler *et al* discovered a case of difference on arm selection preference, at which mir-33* in *Haliotis* expressed 1.56 fold higher than that of miR-33, which implicated the annotation issue of major and minor of mir-33 in *Haliotis*[4]. In this study, we conducted an in-depth analysis on the major and minor forms of miRNAs in medaka. Similar findings were also observed by comparing the copy number of mature miRNAs at both arms of the same precursor (Additional file 1). In summary, we classified these differences on arm selection preference into three classes and used the candidates in Mh set (Additional file 1) for illustration.

**Figure 2:**
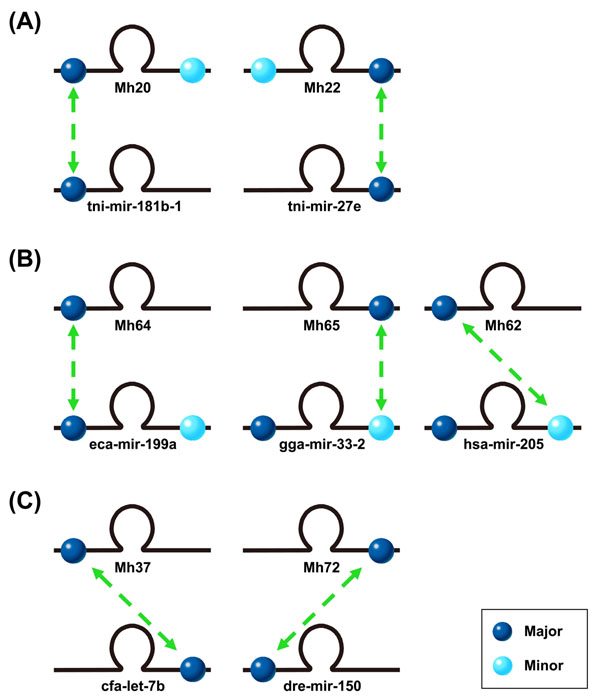
Comparison of arm selection preference of miRNAs within precursor. Dark blue balls and light blue balls denote the location of major form and minor form of mature miRNA, respectively. Double-headed arrows demonstrate sequence conservation between candidate pre-miRNAs and their orthologous ones.

The first class of difference on arm selection preference can be illustrated by Mh20 and Mh22 in Figure 2a. They encode mature miRNAs at both of their 5’ and 3’ arms; however, their orthologous pre-miRNAs, tni-mir-181b-1 and tni-mir-27e, encode mature miRNAs only at 5’ arm or 3’ arm by miRBase annotation. Although we detected mature miRNAs at both arms of Mh20 and Mh22, the major ones of Mh20 and Mh22 are similar to their orthologous pre-miRNAs in terms of sequence and arm selection preference. The minor forms of miRNAs (miRNA stars) can be detected due to the high-throughput of NGS technology. Other candidates, including Mh21, Mh36, Mh43, Mh74, and so on, also demonstrate this phenomenon of this class.

The second class of difference on arm selection preference can be illustrated by Mh64 and Mh65 in Figure 2b. On the contrary to the first class, Mh64 and Mh65 encode mature miRNA only at their 5’ arm or 3’ arm, respectively; nevertheless, their orthologous pre-miRNAs, eca-mir-199a and gga-mir-33-2, encode mature miRNA at both 5’ arm and 3’ arm of the precursors. One possible explanation could just be that the original expression level of the Mh64’s minor one is too low to be detected under such sequencing intensity. However, the 5’ arm and 3’ arm of Mh65 are homologous to gga-miR-33 (major) and gga-miR-33* (minor), respectively. We detected only mature miRNA from 3’ arm of Mh65. Our explanation seemed to fit Mh64 well but not fit Mh65. The better explanation for Mh65 could be that the sequence difference at the loop part of hairpin makes Mh65’s structure differ from gga-mir-33-2’s. Therefore, the altered structure changed the released miRNA/miRNA* duplex and consequently the selection preference of RISC miRNA selection from duplex.

Another interesting example of the second class is Mh62 (Figure 2b) whose orthologous pre-miRNAs is hsa-mir-205. hsa-mir-205 encodes hsa-miR-205 and hsa-miR-205* at its 5’ and 3’ arm, respectively. When a sequence comparison was conducted, we found that the 5’ arm of Mh62 is homologous to the 3’ arm of hsa-mir-205. As a result, the encoded miRNA by Mh62 is homologous to the 3’ arm miRNA of hsa-mir-205 (hsa-miR-205*). Other candidates of this class include Mh50, Mh56, Mh39, and Mh47.

The third class of difference on arm selection preference can be illustrated by Mh37 and Mh72 in Figure 2c. Mh37 and Mh72 encode mature miRNAs individually only at their 5’ arm or 3’ arm; however, their orthologous pre-miRNAs, cfa-let-7b and dre-mir-150, encode mature miRNAs at exactly the opposite arms according to miRBase annotation. Such phenomenon is also observed among known miRNAs in miRBase. For example, ptr-let-7b, mml-let-7b and bta-let-7b encode let-7b only at their 5’ arms; cfa-let-7b encodes let-7b only at its 3’ arm; and, oan-let-7b encodes let-7b and let-7b* at both arms. This observation is similar to Mh62 of second class and shows the conservation between mature miRNA sequences does not guarantee the conservation of the whole precursors. Other candidates in this class include Mh8, Mh18 and Mh12.

### miRNA expression level relation to conservation level

**Figure 3:**
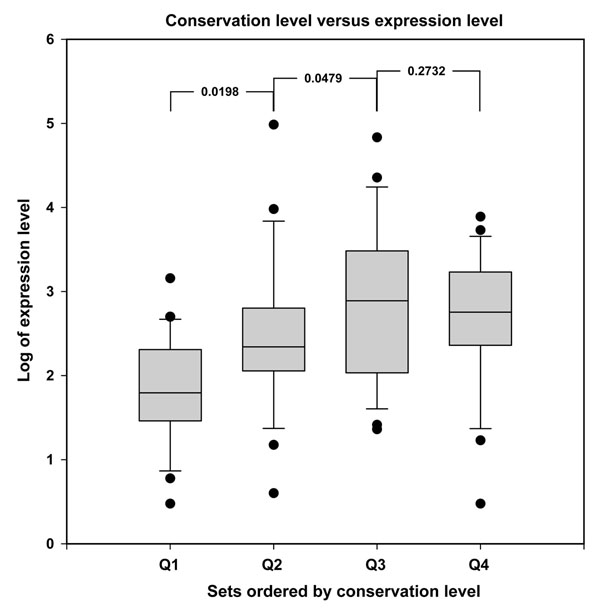
Box plot of expression levels of different sets ordered by conservation level. The plot illustrates that the sets are different from each other in terms of expression level. P-value denotes the result from pair-wised t-test.

It is well accepted that genes involved in basal and important physiological activities are often more conserved during evolution [28]. Owing to selection pressure, they were allowed to have fewer variations and usually showed basal expression level in specific tissue or developmental stage. This could be applied to miRNA genes. Previous studies investigated the relationship between the conservation level and expression level of miRNA genes and concluded that evolutionarily conserved miRNAs were often among the most abundant ones [20,29-31]. In this study, we performed similar assessment on our sequence reads. As shown in Additional file 1, medaka miRNA expression level denotes the copy number of the most abundant isomiR of each miRNA family; conservation level denotes the number of species encoding this particular miRNA family. We evenly divided these 98 miRNA families of Mh set into four quarters, Q1, Q2, Q3 and Q4, by an ascending order of conservation level, followed by plotting the logarithms of expression levels with a box plot. As show in Figure 3, the trend of expression increased upward between sets.

For more detailed comparison, we then did pair-wised t-test analysis on each adjacent pair to test the null hypothesis that the expression levels of the two adjacent sets are the same. As shown in Figure 3, except for Q3Q4 pair, the p-values of all comparison pairs are smaller than 0.05, which rejects the null hypothesis and concludes that the expression levels of different sets are significantly different. In short, the result demonstrates that the more conserved miRNA families tend to have higher expression level, which is consistent with previous report [20].

## Discussion

In the courses of identifying miRNA genes from sequences, there are different strategies depending on whether the target species has known miRNA genes. For the species with known miRNAs registered in miRBase, the mappable reads identical to known miRNAs of own species can be simply regarded as miRNAs. Other mappable reads without known miRNA matches should be carefully evaluated as novel miRNAs [19,20]. On the contrary, for the species without known miRNAs registered in miRBase, all of the mappable reads should be carefully evaluated [4,25]. Since there are no known medaka miRNA reported in miRBase, we applied a SVM pipeline to identify authentic candidate miRNAs.

During our analysis pipeline, there are many reads mapped back to multiple medaka genomic loci even if repeat-masked genome was used. For example, read_1182637 (AACACGAAGCACACACGACGCC), read_7361491 (CCCCCTGCTACATCTACTCCCAGTG) and read_263914823 (TCCGAAAATCCTAAAACGCGC) individually have 45, 50 and 48 occurrences in medaka genome and they were not recognized by RepeatMasker. Therefore, we wonder whether they are truly repeat elements and they were not recognized by RepeatMasker just because they were not included in repbase. Another possible explanation is that such high-frequency elements come from the fact that medaka scaffold assembly quality is less satisfied. In medaka, the total size of genome is about 889 Mb, where 24 chromosomes contribute 717 Mb but 7,164 scaffolds contribute the rest 20% of genome. However, half of the occurrences of seq_1182637, seq_7361491 and seq_263914823 locate at chromosomes and the rest half at scaffolds. With better quality of scaffold assembly, such multiple-loci problem could be solved.

In this study, we found that highly conserved miRNA families seem to have higher expression levels. There are many genes, such GAPDH, acting basal and critical functions so that their roles can not be replaced, which results in their conservation in the course of evolution. Similar conclusions can be made on these conserved miRNA families and implies their critical and un-replaceable roles in medaka. In fact, performing such examination on miRNAs sampled from single tissue, organ, development stage or cell line might cause bias because the miRNA’s expression profile in different tissues or stages are usually distinct. Our RNA samples were collected from whole body of one pair of male and female medaka so that our result to such issue would be reliable and more unbiased.

During miRNA maturation, there is an arm selection preference of mature miRNAs within precursors. In this study, we investigated the differences on arm selection preference. The results from the third class of difference demonstrates the conservation between mature miRNA sequences does not guarantee the conservation of the whole precursors. In miRNA gene identification studies, sequence conservation was a commonly used criterion of some bioinformatics pipelines to identify homologous miRNA in genomes [32-34]. It might be too stringent to demand the conservation of the whole hairpin in miRNA identification studies [35]. On the contrary, it is more suitable to demand the conservation of the mature miRNA.

## Conclusion

Medaka is a widely used model animal and usually involved in many biomedical studies, including the ones on development biology [36]. miRNAs were also reported to play important regulation roles during animal embryo development [37]. Therefore, identifying medaka miRNA genes may contribute to the studies on animal development and provide insight into the regulation on development.

## Materials and methods

### Raw reads from SOLiD platform

Two healthy adult medaka fish (*Oryzias latipes*), one male and one female, were provided by Dr. Pung-Pung Hwang (Institute of Cellular and Organismic Biology, Academia Sinica). They were lysed with a tissue lyser (TissueLyser QIAGEN), followed by RNA extraction with Trizol reagent (Invitrogen) according to the manufacturer’s protocol. Total RNA from the whole bodies of male and female medaka fish was thus pooled and used for small RNA direct sequencing analysis by ABI SOLiD system.

### Mapping pipeline

The unique reads with copy number equal to or greater than three were processed for trimming adaptor, followed by being mapped back to genome by Razers program [38]. Owing to the concern of repeat element, we used repeat-masked genomes for mapping and the release versions of genomes for medaka is oryLat2, downloaded from UCSC. In this study, we requested a mappable read must completely identical to genome loci without mismatch or gap as previous report [19]. In addition, a mappable read must range from 18 to 25 nt. in length.

### Excluding sequence reads from protein-coding genes and repeat elements

Owing to the concern of contamination by protein-coding gene or other ncRNAs, candidate miRNAs highly homologous to RefSeq sequences were usually removed in miRNA gene identification studies [19]. Up to now, there are only 474 medaka protein-coding reference genes reported in RefSeq release 37. Therefore, all mappable sequence reads with more than 90% identity to any reference sequence (from RefSeq 37) were discarded. Besides, we also had these mappable reads processed by RepeatMasker to exclude repeat elements. However, we still observed many reads with multiple mappable genomic loci (see Discussion). For solving such problem, sequence reads mappable back to more than ten genomic loci were also discarded in this study.

### miRNA identification by SVM pipeline

The sequences of the genomic loci mapped back by reads were considered as candidate miRNAs. For each candidate miRNA, we extended 60-nt regions individually at its upstream and downstream and the resulting ~140 nt. fragments were considered as candidate pre-miRNAs. Each pair of candidate miRNA and pre-miRNA was subject to folding and calculation of the ten classification features, followed by classified by our SVM pipeline for identifying authentic miRNAs. The SVM pipeline classifies input cases into positive or negative set according to the trained model [24].

## Authors’ contributions

SCL performed and executed this study and wrote the draft of this manuscript. WCC and CNH were responsible for Construction of SVM classification model. LYH and CHL helped tissue preparation and RNA extraction. PPH provided medaka for experiment. WCL supervised the study and edited the manuscript.

## Competing interests

The authors declare that they have no competing interests.

## Supplementary Material

Additional file 1 Candidate miRNAs information of Mh set. Each isomiR of candidate pre-miRNAs was tagged with copy number and p-value. Click here for file

Additional file 2 Candidate miRNAs information of Mn set. Each isomiR of candidate pre-miRNAs was tagged with copy number and p-value. Click here for file

Additional file 3 miRNA cluster information. Click here for file
