# Supplementary material for: Discovery and characterization of medaka miRNA genes by next generation sequencing platform
Source: BMC Genomics. 2010 Dec 2;11(Suppl 4):S8. doi: 10.1186/1471-2164-11-S4-S8 (PMC3005926; doi:10.1186/1471-2164-11-S4-S8)
Supplement: Additional file 3 — miRNA cluster information. [file 1471-2164-11-S4-S8-S3.doc]

**Additional File 3**

| **Cluster ID** | **Candidate ID** | **Chrom.** | **strand** | **start pos.** |
| --- | --- | --- | --- | --- |
| 1 | Mn11 | chr2 | + | 30824943 |
| 1 | Mn12 | chr2 | + | 30825474 |
| 1 | Mn13 | chr2 | + | 30826749 |
| 1 | Mn14 | chr2 | + | 30829578 |
| 2 | Mh6 | chr2 | - | 28254368 |
| 2 | Mh7 | chr2 | - | 28254507 |
| 3 | Mh8 | chr2 | - | 30826142 |
| 3 | Mn17 | chr2 | - | 30829254 |
| 4 | Mn18 | chr3 | + | 4273945 |
| 4 | Mn19 | chr3 | + | 4275207 |
| 5 | Mh10 | chr3 | + | 15107307 |
| 5 | Mh11 | chr3 | + | 15107452 |
| 5 | Mh12 | chr3 | + | 15107614 |
| 5 | Mh13 | chr3 | + | 15107783 |
| 5 | Mh14 | chr3 | + | 15108084 |
| 6 | Mh22 | chr4 | + | 34610548 |
| 6 | Mh23 | chr4 | + | 34612920 |
| 7 | Mh27 | chr5 | + | 11768469 |
| 7 | Mh28 | chr5 | + | 11768783 |
| 8 | Mh36 | chr6 | + | 6146863 |
| 8 | Mh37 | chr6 | + | 6147325 |
| 9 | Mh42 | chr6 | - | 16407304 |
| 9 | Mh43 | chr6 | - | 16407646 |
| 10 | Mh46 | chr7 | + | 6519353 |
| 10 | Mh47 | chr7 | + | 6519816 |
| 11 | Mh50 | chr7 | + | 13613915 |
| 11 | Mh51 | chr7 | + | 13614181 |
| 12 | Mh53 | chr7 | + | 23588373 |
| 12 | Mh54 | chr7 | + | 23588631 |
| 13 | Mh56 | chr7 | - | 217696 |
| 13 | Mh57 | chr7 | - | 217985 |
| 14 | Mh69 | chr8 | - | 13687323 |
| 14 | Mh70 | chr8 | - | 13688175 |
| 14 | Mh71 | chr8 | - | 13688406 |
| 15 | Mh74 | chr9 | + | 27956541 |
| 15 | Mh75 | chr9 | + | 27956674 |
| 16 | Mh76 | chr9 | - | 8309654 |
| 16 | Mh77 | chr9 | - | 8313668 |
| 17 | Mn57 | chrM | + | 46 |
| 17 | Mn58 | chrM | + | 173 |
| 17 | Mn59 | chrM | + | 334 |
| 17 | Mn60 | chrM | + | 442 |
| 17 | Mn61 | chrM | + | 2806 |
| 17 | Mn62 | chrM | + | 2920 |
| 17 | Mn63 | chrM | + | 5014 |
| 17 | Mn64 | chrM | + | 6870 |
| 17 | Mn65 | chrM | + | 8728 |
| 18 | Mh83 | chr10 | + | 2001187 |
| 18 | Mh84 | chr10 | + | 2001485 |
| 19 | Mh90 | chr11 | + | 21880298 |
| 19 | Mh91 | chr11 | + | 21880471 |
| 20 | Mn83 | chr13 | + | 17166411 |
| 20 | Mn84 | chr13 | + | 17166557 |
| 21 | Mh112 | chr13 | + | 32654589 |
| 21 | Mh113 | chr13 | + | 32654974 |
| 22 | Mn88 | chr13 | - | 21201854 |
| 22 | Mn89 | chr13 | - | 21202737 |
| 22 | Mn90 | chr13 | - | 21203397 |
| 22 | Mn91 | chr13 | - | 21203542 |
| 23 | Mn94 | chr13 | - | 31014038 |
| 23 | Mn95 | chr13 | - | 31014152 |
| 23 | Mn96 | chr13 | - | 31014222 |
| 24 | Mh122 | chr14 | + | 20215672 |
| 24 | Mh123 | chr14 | + | 20215822 |
| 25 | Mh126 | chr14 | - | 1144184 |
| 25 | Mh127 | chr14 | - | 1150882 |
| 25 | Mh128 | chr14 | - | 1151199 |
| 26 | Mh130 | chr14 | - | 15646480 |
| 26 | Mh131 | chr14 | - | 15650352 |
| 26 | Mh132 | chr14 | - | 15650826 |
| 27 | Mh140 | chr16 | + | 3867442 |
| 27 | Mh141 | chr16 | + | 3873569 |
| 27 | Mn112 | chr16 | + | 3874736 |
| 28 | Mn113 | chr16 | + | 5367820 |
| 28 | Mn114 | chr16 | + | 5368082 |
| 29 | Mh147 | chr17 | + | 16117948 |
| 29 | Mh148 | chr17 | + | 16119235 |
| 30 | Mh155 | chr17 | - | 29407313 |
| 30 | Mh156 | chr17 | - | 29410125 |
| 31 | Mh157 | chr17 | - | 29776748 |
| 31 | Mh158 | chr17 | - | 29779201 |
| 31 | Mh159 | chr17 | - | 29781101 |
| 32 | Mn136 | chr18 | + | 6475147 |
| 32 | Mn137 | chr18 | + | 6479448 |
| 33 | Mn144 | chr18 | + | 25352177 |
| 33 | Mn145 | chr18 | + | 25353610 |
| 34 | Mn148 | chr18 | - | 12519891 |
| 34 | Mn149 | chr18 | - | 12520251 |
| 34 | Mn150 | chr18 | - | 12521460 |
| 35 | Mn161 | chr20 | + | 9957356 |
| 35 | Mn162 | chr20 | + | 9959155 |
| 36 | Mh176 | chr21 | - | 25601297 |
| 36 | Mh177 | chr21 | - | 25601991 |
| 36 | Mh178 | chr21 | - | 25602002 |
| 37 | Mh179 | chr21 | - | 25693131 |
| 37 | Mh180 | chr21 | - | 25693239 |
| 37 | Mh181 | chr21 | - | 25693363 |
| 37 | Mh182 | chr21 | - | 25693520 |
| 37 | Mh183 | chr21 | - | 25693655 |
| 37 | Mh184 | chr21 | - | 25694055 |
| 38 | Mn177 | chr23 | + | 6723344 |
| 38 | Mn178 | chr23 | + | 6724979 |
| 39 | Mh190 | chr23 | + | 8740355 |
| 39 | Mh191 | chr23 | + | 8740492 |
| 40 | Mh193 | chr23 | + | 14604483 |
| 40 | Mh194 | chr23 | + | 14604891 |
| 41 | Mh199 | chr24 | + | 13641157 |
| 41 | Mh200 | chr24 | + | 13641851 |
| 42 | Mn183 | chr24 | - | 23293421 |
| 42 | Mn184 | chr24 | - | 23295770 |
| 43 | Mh204 | scaffold617 | + | 68781 |
| 43 | Mh205 | scaffold617 | + | 68829 |
| 43 | Mn198 | scaffold617 | + | 68911 |
| 44 | Mn213 | scaffold1172 | - | 29113 |
| 44 | Mn214 | scaffold1172 | - | 30466 |
| 45 | Mn248 | scaffold2480 | - | 4642 |
| 45 | Mn249 | scaffold2480 | - | 4797 |
| 45 | Mn250 | scaffold2480 | - | 5540 |
| 45 | Mn251 | scaffold2480 | - | 8402 |
| 45 | Mn252 | scaffold2480 | - | 9243 |
| 45 | Mn253 | scaffold2480 | - | 10984 |
| 46 | Mn255 | scaffold2736 | - | 3315 |
| 46 | Mn256 | scaffold2736 | - | 4081 |
| 47 | Mn261 | scaffold2906 | - | 2145 |
| 47 | Mn262 | scaffold2906 | - | 9575 |
| 47 | Mn263 | scaffold2906 | - | 9646 |
| 47 | Mh219 | scaffold2906 | - | 10766 |
| 47 | Mh220 | scaffold2906 | - | 11181 |
| 48 | Mn264 | scaffold2920 | - | 3415 |
| 48 | Mn265 | scaffold2920 | - | 3491 |
| 48 | Mn266 | scaffold2920 | - | 3547 |
| 48 | Mn267 | scaffold2920 | - | 3605 |
| 48 | Mh221 | scaffold2920 | - | 6355 |
| 48 | Mn268 | scaffold2920 | - | 6417 |
| 48 | Mh222 | scaffold2920 | - | 6708 |
| 48 | Mn269 | scaffold2920 | - | 6963 |
| 48 | Mh223 | scaffold2920 | - | 7123 |
| 48 | Mh224 | scaffold2920 | - | 8204 |
| 48 | Mn270 | scaffold2920 | - | 8425 |
| 48 | Mh225 | scaffold2920 | - | 8619 |
| 49 | Mn273 | scaffold3303 | + | 2410 |
| 49 | Mh226 | scaffold3303 | + | 4952 |
| 49 | Mn274 | scaffold3303 | + | 5551 |
| 50 | Mn281 | scaffold3840 | - | 339 |
| 50 | Mn282 | scaffold3840 | - | 7114 |
| 51 | Mh231 | scaffold3863 | + | 52 |
| 51 | Mn283 | scaffold3863 | + | 193 |
| 51 | Mn284 | scaffold3863 | + | 472 |
| 51 | Mn285 | scaffold3863 | + | 638 |
| 51 | Mn286 | scaffold3863 | + | 788 |
| 51 | Mn287 | scaffold3863 | + | 2032 |
| 51 | Mh232 | scaffold3863 | + | 2800 |
| 52 | Mn302 | scaffold5089 | + | 85 |
| 52 | Mn303 | scaffold5089 | + | 725 |
| 52 | Mn304 | scaffold5089 | + | 821 |
| 52 | Mn305 | scaffold5089 | + | 915 |
| 52 | Mh233 | scaffold5089 | + | 1364 |
| 52 | Mn306 | scaffold5089 | + | 1559 |
| 52 | Mn307 | scaffold5089 | + | 2376 |
| 52 | Mn308 | scaffold5089 | + | 2662 |
| 52 | Mh234 | scaffold5089 | + | 2730 |
| 52 | Mn309 | scaffold5089 | + | 2965 |
| 52 | Mn310 | scaffold5089 | + | 3410 |
| 52 | Mn311 | scaffold5089 | + | 3573 |
| 52 | Mn312 | scaffold5089 | + | 3699 |
| 52 | Mh235 | scaffold5089 | + | 3795 |
| 52 | Mh236 | scaffold5089 | + | 4058 |
| 52 | Mh237 | scaffold5089 | + | 4453 |
| 53 | Mn313 | scaffold5234 | + | 2629 |
| 53 | Mh238 | scaffold5234 | + | 2692 |
| 54 | Mn315 | scaffold6261 | - | 2952 |
| 54 | Mn316 | scaffold6261 | - | 3101 |
| 55 | Mn327 | scaffold8092 | - | 16 |
| 55 | Mh240 | scaffold8092 | - | 348 |
| 56 | Mn328 | ultracontig1 | - | 320211 |
| 56 | Mh241 | ultracontig1 | - | 320316 |
| 56 | Mn329 | ultracontig1 | - | 321824 |
| 57 | Mn332 | ultracontig37 | + | 112308 |
| 57 | Mn333 | ultracontig37 | + | 112715 |
| 58 | Mn334 | ultracontig62 | + | 322800 |
| 58 | Mh244 | ultracontig62 | + | 322987 |
| 59 | Mh248 | ultracontig115 | + | 5319909 |
| 59 | Mh249 | ultracontig115 | + | 5320131 |
| 60 | Mn343 | ultracontig222 | + | 534129 |
| 60 | Mn344 | ultracontig222 | + | 534985 |
| 60 | Mh252 | ultracontig222 | + | 535163 |
| 60 | Mh253 | ultracontig222 | + | 536161 |
| 60 | Mn345 | ultracontig222 | + | 536351 |
| 61 | Mh196 | chr23 | - | 6185677 |
| 61 | Mh197 | chr23 | - | 6186600 |
| 62 | Mh202 | scaffold357 | - | 438972 |
| 62 | Mh203 | scaffold357 | - | 439006 |
| 63 | Mh217 | scaffold2659 | - | 10145 |
| 63 | Mh218 | scaffold2659 | - | 10258 |
